# Not to be overlooked: dipeptides and their role in plant stress resilience

**DOI:** 10.1093/jxb/eraf311

**Published:** 2025-07-09

**Authors:** Pallavi Agarwal, Hillary D Fischer, Maria D Camalle, Aleksandra Skirycz

**Affiliations:** Michigan State University, East Lansing, MI 48823, USA; Michigan State University, East Lansing, MI 48823, USA; Michigan State University, East Lansing, MI 48823, USA; Michigan State University, East Lansing, MI 48823, USA; Bielefeld University, Germany

**Keywords:** Cyclic dipeptides, dipeptides, γ-glutamyl dipeptides, plant growth, stress resilience

## Abstract

Dipeptides are structurally diverse small molecules with varying modes of biogenesis and function. In plants, dipeptides were historically associated with nitrogen storage and mobilization; however, they are also reported to act as antioxidants, signaling molecules, protein regulators, and modulators of microbial communities. In this review, we discuss the structural diversity and biogenesis of dipeptides, with respect to (i) proteinogenic dipeptides that are products of protein degradation, (ii) non-proteinogenic amino acid dipeptides, such as those containing a γ-glutamyl group, and (iii) cyclic dipeptides, largely derived from microorganisms. Highlighted are recent examples of dipeptides that were shown to display plant health-promoting activities, including boosting growth and improving stress resilience against pathogens, salinity, chilling, and heat, making the case that these overlooked compounds are more than what meets the eye.

## Teaser

Dipeptides are bioactive compounds with relevance across domains of life. This review consolidates and questions our current understanding of their biogenesis and diverse functionality in plants.

## Introduction

Peptides are central to numerous physiological processes, extensively studied for their regulatory functions. In contrast, the shortest of peptides, dipeptides—composed of just two amino acids—have received comparatively less attention, despite emerging evidence suggesting distinct roles and bioactivities. Considering only proteinogenic amino acids, there are 400 dipeptides, but the number of dipeptide compounds is much larger, as the dipeptides can also contain non-proteinogenic amino acids. Moreover, linear dipeptides can undergo cyclization to form cyclic dipeptides or 2,5-diketopiperazines (DKPs), contributing to their vast structural and functional diversity. While some dipeptides derive from protein degradation, others such as γ-glutamyl dipeptides have dedicated biosynthetic pathways. Dipeptides can be found across life kingdoms and are considered ancient compounds whose presence may date back to the primordial soup wherein life initially evolved (Canavelli *et al.*, 2019). In animals, dipeptides have been shown to display various health-promoting activities, including anti-cancer, nociceptive, anti-inflammatory, and neuroprotective (Vázquez-Rivera *et al*., 2015; Bojarska *et al.*, 2021). Dipeptides are also crucial for inter-organismal communication and signaling in microbes, shaping not only human microbiomes but also plant–microbial interactions (Nishioka *et al.,* 2022; Solis-Ortiz *et al*., 2022). Despite all the above and with some exceptions, dipeptides remain relatively understudied, especially in plants. For the purpose of this review, we have divided sections into linear proteinogenic and non-proteinogenic dipeptides and cyclic dipeptides. Here we address the important aspects of how dipeptides accumulate and exert their function, highlighting the key knowledge gaps in identification, biogenesis, and function.

## Proteinogenic dipeptides—more than merely proteolytic intermediates

**Fig. 1.**
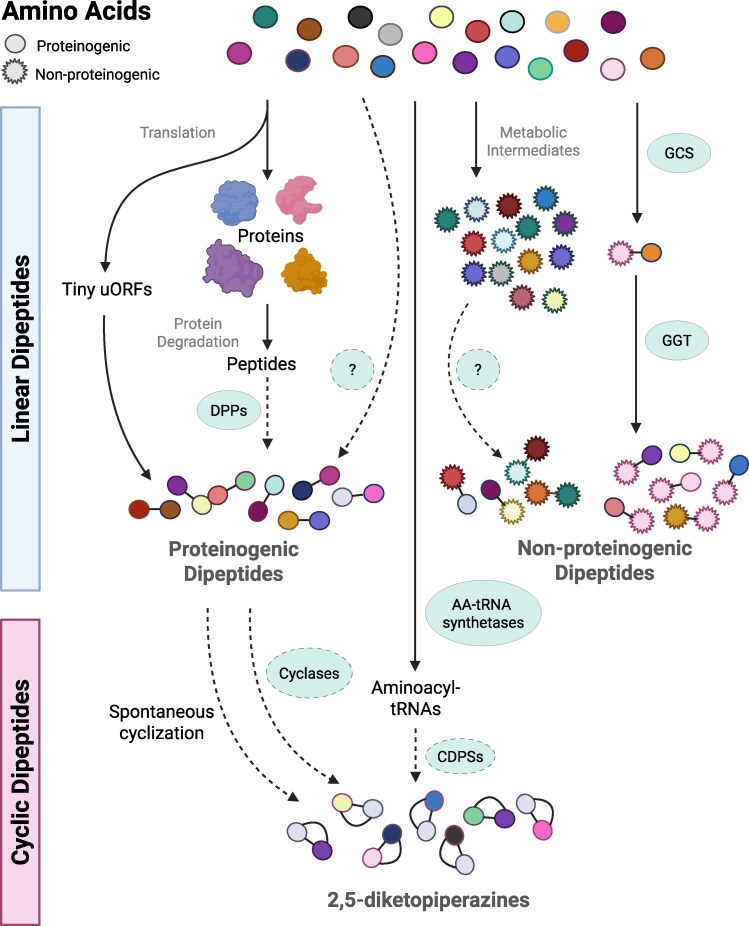
Model of dipeptide formation in plants. Naturally occurring dipeptides are here categorized as proteinogenic and non-proteinogenic linear dipeptides and cyclic dipeptides. In the plant, proteinogenic dipeptides are known to be generated during protein degradation, where short peptides are cleaved by dipeptidyl peptidases (DPPs), including organellar oligopeptidase, cathepsin B, and Nudix hydrolase 3. They can also be generated during protein translation in the form of tiny uORFs or possibly directly synthesized from their amino acids. Non-proteinogenic amino acids are, generally speaking, metabolic intermediates, and are not incorporated into proteins. The only known non-proteinogenic dipeptides in plants are γ-glutamyl dipeptides synthesized during glutathione cycling from γ-glutamylcysteine synthetase (GCS) which forms γ-glutamylcysteine. The γ-glutamyl can then be moved to a donor amino acid by γ-glutamyltransferase (GGT), giving rise to a plethora of γ-glutamyl dipeptides. Currently, there are no documented mechanisms for formation of 2,5-diketopiperazines (DKPs) in plants. Nevertheless, we hypothesize that this formation may result from the cyclization of aminoacyl-tRNAs from proteinogenic amino acids, which are cyclized by cyclic dipeptidase synthetases (CDPSs) similar to those found in bacteria, or through the spontaneous cyclization of linear dipeptides, as occurs in mammalian cells. Solid lines indicate known components of pathways, and dashed lines represent hypothesized pathways or enzymes. This figure was created in BioRender. Skirycz, A. (2025) https://BioRender.com/0029fe3.

Protein degradation—whether mediated by the ubiquitin–proteasome system (UPS), vacuolar pathways such as autophagy (Dikic, 2017), or protease complexes in plastids and mitochondria (Kmiec *et al.*, 2014; Rowland *et al.*, 2022)—first generates short peptides, which are further cleaved by a suite of diverse endo- and exopeptidases (Fig. 1). The latter operate on terminal peptide bonds, releasing amino acids, dipeptides, or tripeptides (Isono *et al*., 2024). Dipeptidyl peptidases (DPPs) act on short peptides to generate dipeptides and, in plants, the few DPPs that have been characterized include organellar oligopeptidase (Kmiec *et al*., 2013), cathepsin B (Porodko *et al*., 2018), and Nudix hydrolase 3 (Karačić *et al*., 2017), pointing to the need for more research in this field. Karačić *et al*., 2017), pointing to the need for more research in this field. The released dipeptides are then degraded to amino acids by dipeptidases such as prolyl aminopeptidases (PAP1 and PAP2) which cleave proline from the N-terminus of short peptides (Ghifari *et al.*, 2022). Beyond proteolytic origins, a recent report points to alternative biosynthetic pathways. A ribosome profiling study in Arabidopsis identified 2921 tiny upstream ORFs (uORFs), including 388 coding for N-terminal methionine dipeptides, demonstrating that at least some dipeptides can be synthesized directly during translation (Wu *et al.*, 2024). Other possibilities include enzymatic synthesis directly from amino acids by specialized or non-ribosomal peptide-like synthetases, which so far remain unidentified in plants (Fig. 1). Support for selective biosynthesis and accumulation comes from Manabe (1992), who found that rice plants fed with D-Ala accumulated D-Ala-containing dipeptides. These findings suggest that dipeptide production and accumulation in plants may be more diverse and more regulated than previously recognized.

Exopeptidases and dipeptidases vary in cleavage specificity, probably contributing to the reported and often specific dipeptide accumulation patterns. For example, Asp- or Glu-containing dipeptides increase under heat and dark conditions but decline with cold exposure in Arabidopsis (Thirumalaikumar *et al.*, 2021). Similarly, Pro-containing dipeptides correlate with diurnal carbon status (Calderan-Rodrigues *et al.*, 2021). Such differential accumulation has also been observed across Arabidopsis root cell types (Moussaieff *et al.*, 2013), during leaf development (Omidbakhshfard *et al.*, 2021) and seed germination (Tiozon *et al.*, 2023), and in response to microbial infection (Strehmel *et al.*, 2016, 2017), alkali stress (Wang *et al.*, 2024), and drought (L. Yang *et al.*, 2018). Interestingly, a recent study on tomatoes found a positive association between dipeptide accumulation and recovery from drought in the plant following the foliar application of certain protein hydrolysates (Leporino *et al.*, 2024). Moreover, specific dipeptides have also been detected in root exudates (Strehmel *et al.*, 2017; Wang *et al.*, 2024) suggesting their role in plant–microbe interactions. Such context-dependent accumulation, as well as presence in the root exudates, raises questions about the mechanisms underlying dipeptide distribution within the plant. The efficient transport of dipeptides is associated with the evolutionarily conserved dipeptide transporters that belong to the proton-coupled peptide transporters (NTR1/PTR) family (Ito *et al.*, 2013). In Arabidopsis, four confirmed and two more putative transporters vary in their subcellular localization and expression patterns (Steiner *et al*., 1994; Komarova *et al.*, 2008; Weichert *et al.*, 2012; Choi *et al.*, 2020). Dipeptide transport is associated with the nitrogen reallocation between nitrogen source and sink organs (Tegeder and Rentsch, 2010)—most notably from senescing leaves to flowers and seeds, and from seed endosperm to germinating seedlings. In animals, dipeptide transport from the gut lumen crosstalks with target of rapamycin (TOR) signaling pathways (Daniel *et al*., 2006). A recent study in rice found that water-saving treatments negatively impact the yield and nitrogen use efficiency of the paddy-field rice due to the compromised TOR signaling that suppresses the translation of the dipeptide transporter NPF7.3 (Li *et al.*, 2024). However, this transporter is also associated with auxin transport (Watanabe *et al.*, 2020), leaving the involvement of dipeptides in TOR signaling ambiguous. These findings open up new avenues of research into dipeptides acting not only as mobile nitrogen stores but also as potential modulators of TOR signaling.

In addition to their roles in nitrogen transport and metabolism, the proteinogenic dipeptides were found interacting and regulating the activity of proteins in a dipeptide-specific manner (Veyel *et al.*, 2017, 2018; Wagner *et al.*, 2025). For instance, Tyr–Asp binds and inhibits a glycolytic enzyme, glyceraldehyde-3-phosphate dehydrogenase 1/2 (GAPC1/2), leading to changes in carbon flux and steady-state metabolite levels. Plant supplementation with 100 µM Tyr–Asp shifted glycolytic flux towards the pentose phosphate pathway (PPP) and NADPH production (Moreno *et al.*, 2021), and reduced growth penalty associated with salt and oxidative stress. These findings were validated in commercial fruit varieties, where exogenous 1.5–4.5 mM Tyr–Asp treatment delayed fruit senescence in litchi (Zhiyan *et al.*, 2025) and mitigated chilling stress in bananas (Zhu *et al.*, 2025). The authors reported an increased NADPH/NADP^+^ ratio and glutathione (GSH) level, along with higher activity of reactive oxygen species- (ROS) scavenging enzymes. Catalase (CAT), superoxide dismutase (SOD), and peroxidase (POD) activities were all augmented by Tyr–Asp supplementation, which was related to the increase in gene expression levels (see Box 1A).

Box 1. **Key developments in the biogenesis and functionality of dipeptides in plants**

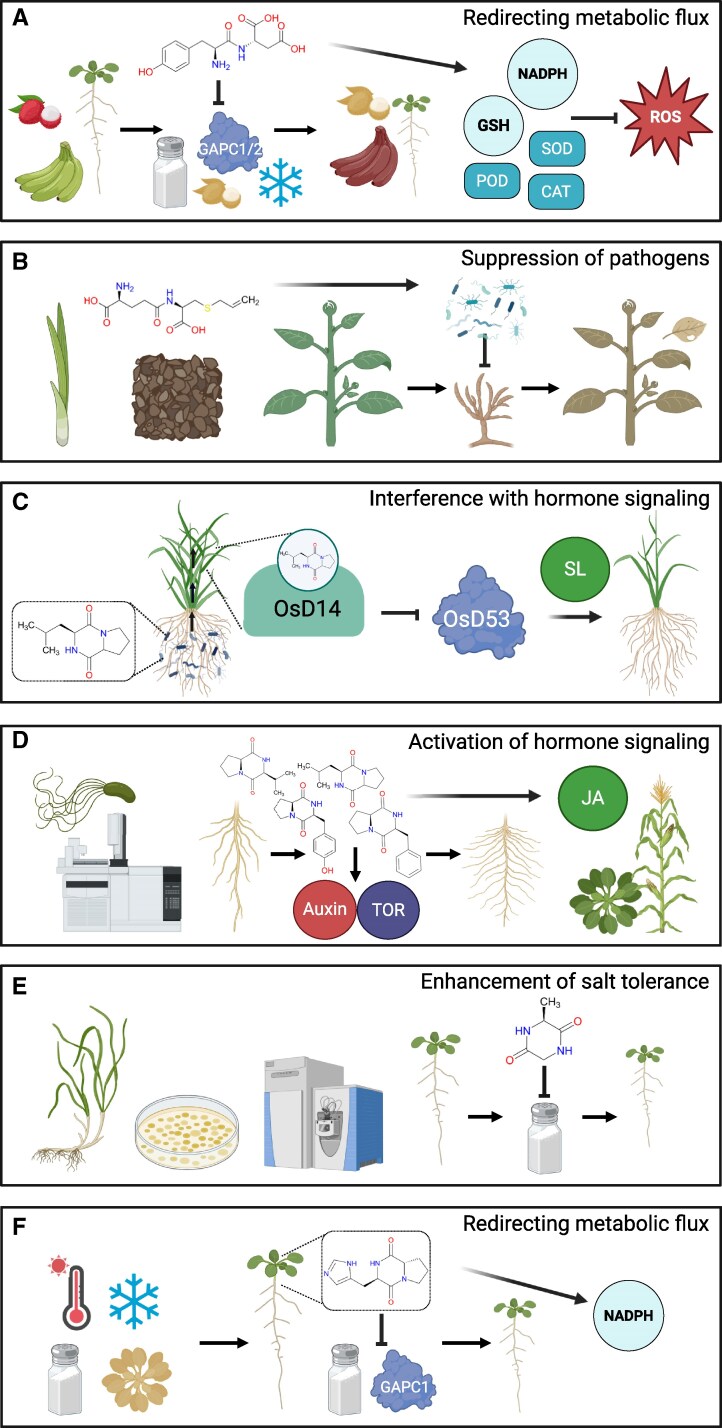

This schematic represents a selection of recent studies linking various dipeptides to plant growth and stress response.

(A) Zhu *et al.* (2025) reported that Tyr–Asp application to banana fruits protects them from chilling stress by increasing the NAPDH/NADP^+^ ratio and augmenting the activity of antioxidant enzymes. Further, Zhiyan *et al.* (2025) also established delayed senescence in litchi following the same mechanism. These studies corroborate previous work in Arabidopsis by Moreno *et al*. (2021) which demonstrated the role of Tyr–Asp in redirecting glycolytic flux toward the pentose phosphate pathway and NADPH production.

(B) Nishioka *et al.* (2022) demonstrated that γ-glutamyl-*S*-allyl-cysteine (GSAC) amendment to the soil suppresses Fusarium wilt severity in cucumber and spinach. The suppressive activity was linked to the GSAC-associated change in the soil microbe community.

(C) Zhang *et al.* (2025) elucidated the mechanism by which bacterially synthesized cyclo(L-Leu–L-Pro) inhibits tiller overgrowth by activating the strigolactone (SL) signaling pathway.

(D) Solis-Ortiz *et al*. (2022) reported the role of bacterial proline-containing DKPs in eliciting plant immune responses and activating jasmonic acid (JA) signaling. This work complements previous studies demonstrating the role of DKPs in lateral root growth induction via auxin and TOR signaling in maize and Arabidopsis (Ortiz-Castro *et al.*, 2011; González *et al*., 2017; Corona-Sánchez *et al.*, 2019; González-López *et al.*, 2021).

(E) Hung *et al*. (2024) identified and characterized cyclo(L-Ala–L-Gly) from an endophytic microbe, *Priestia megaterium* BP01R2, which is associated with the salt marsh plant *Bolboschoenus planiculmis.* Furthermore, cyclo(L-Ala–L-Gly) was shown to alleviate salinity stress in Arabidopsis.

(F) Minen *et al.* (2025) identified and characterized cyclo(L-His–L-Pro) in Arabidopsis, which accumulated upon abiotic stresses, and the accumulation was developmental stage-specific. Cyclo(L-His–L-Pro) inhibited GAPC1 activity, which in turn increased the NAPDH/NADP^+^ ratio and conferred salt stress resilience.

This figure was created in BioRender. Skirycz, A. (2025) https://BioRender.com/n2mdsch.

A different enzyme whose activity is inhibited by Ala–Ile, but not Tyr–Asp, is phosphoenolpyruvate carboxykinase 1 (PEPCK1) (Moreno *et al.*, 2021). PEPCK1 is involved in the mobilization of lipid reserves during early seedling establishment that ties in nicely with dipeptide accumulation during seed germination (Tiozon *et al.*, 2023). Of note, Ala-containing dipeptides, the byproducts of corn gluten hydrolysate, effectively inhibited root growth of germinating grass seeds, which may be connected to the effect on the PEPCK1 activity (Liu and Christians, 1994). Further experiments to identify and characterize the function and the mode of action of different dipeptides in plants are required to build a better understanding of dipeptides, enabling them to be a tool for crop improvement.

## Non-proteinogenic dipeptides—made for more

In contrast to proteinogenic dipeptides, the formation of dipeptides with at least one non-proteinogenic amino acid occurs via dedicated enzymes (Fig. 1). In plants, dipeptides containing γ-glutamyl are the only group of non-proteinogenic dipeptides reported to date. Their biosynthesis is largely attributed to GSH cycling and is indeed tightly linked with fluctuations in GSH. The pathway begins with γ-Glu–Cys synthetase (GCS) combining L-Glu and L-Cys to make γ-Glu–Cys, and is the rate-limiting enzyme in GSH synthesis (Wang *et al.*, 2017). When GCS is overexpressed, leading to increased γ-Glu–Cys, it confers tolerance to heavy metals, such as cadmium (He *et al.*, 2015; Zhang *et al.*, 2022; Chhikara *et al.*, 2024), as well as to abiotic stressors, such as drought and salt (Choe *et al.*, 2013; Yang *et al.*, 2024). However, this may also be from an increase in GSH only, as γ-Glu–Cys is almost immediately converted to GSH (Noctor *et al*., 1997).

Further γ-glutamyl dipeptides are synthesized in the plant by different enzymes in GSH metabolism, such as γ-glutamyl transpeptidase (GGT), which can transfer a γ-glutamyl residue from GSH to another proteinogenic or non-proteinogenic amino acid (Shaw *et al.*, 2005). γ-glutamyl dipeptides have been found in several plants, and GGT is present in higher functioning plants. Specifically, they have been detected in legume seeds (Li *et al.*, 2025), where they are thought to act as nitrogen stores. However, they probably serve more roles in plants, similar to linear proteinogenic dipeptides. For example, γ-glutamyl-*S*-allyl-cysteine (GSAC), found in *Allium*, is reportedly a plant defense compound with microbial modification activities (see Box 1B). In a recent study, GSAC amendment to the soil was shown to suppress Fusarium wilt severity in cucumber and spinach through GSAC-induced changes in the structure of the soil microbe community (Nishioka *et al.*, 2022). Additionally, the stress response of roots from pumpkin seedlings treated with tetracycline was marked by an increase in γ-Glu–Ala and GSH, and a decrease in γ-Glu–Cys (Yu *et al.*, 2024). The authors suspected γ-Glu–Ala to be an antioxidant, and indeed dipeptides such as γ-Glu–Cys and γ-Glu–Met that contain sulfur residues act as excellent ROS scavengers. However, whether γ-Glu–Ala, containing only methyl side chains, may act as a scavenger is questionable, but it may provide indirect support for redox regulation.

The question remains of whether γ-glutamyl dipeptides have regulatory roles in metabolism similar to proteinogenic dipeptides. In animals, γ-glutamyl dipeptides have been extensively studied in protein regulation (reviewed in Li *et al.*, 2025). Many of the γ-glutamyl dipeptides present in plants, including γ-Glu–Cys, γ-Glu–Met, γ-Glu–Leu, γ-Glu–Phe, and γ-Glu–Tyr, act as extracellular calcium-sensing receptor agonists (Ohsu *et al.*, 2010) and competitively inhibit dipeptidyl peptidase-IV ([Bibr eraf311-B75][Bibr eraf311-B47]) and competitively inhibit dipeptidyl peptidase-IV (J. Yang *et al.*, 2018). J. Yang *et al.*, 2018). With respect to plants, there are no known receptors for γ-Glu dipeptides. However, γ-Glu–Glu partially activates the neuronal *N*-methyl-D-aspartate receptors in humans and rats (Sebih *et al.*, 2022), a homolog of glutamate receptor-like (*GLR*) genes in plants which can be activated by glutamate, as well as other ligands (Qi *et al.*, 2006; Mousavi *et al.*, 2013). Indeed, even GSH acts as a GLR agonist. In plants, GLRs are involved not only in stress response, but also in root development, pollen tube growth, seedling germination, and stomatal regulation (Yu *et al.*, 2022). These findings together warrant further investigation into γ-glutamyl dipeptides in plant protein–metabolite interactions.

In plants, GSH cycling occurs via two pathways. GGT was originally thought to be involved in the main pathway for GSH turnover in all cellular compartments; however, studies in Arabidopsis and barley found it to be involved largely in cycling of GSH in the apoplast, with a GGT-independent pathway functioning within the cytosol (Martin *et al.*, 2007; Ohkama-Ohtsu *et al.*, 2008; Ferretti *et al.*, 2009). In apoplastic GSH cycling, breakdown products, including γ-glutamyl dipeptides, are transported back into the cell to be further processed. It is unknown by which means γ-glutamyl dipeptides are transported back into the cytosol; however, recently Dubey *et al*. (2025) demonstrated that γ-Glu–Met transport across the plasma membrane in *Saccharomyces cerevisiae* is done with a highly specific transporter, Seo1p. The authors found that even n-Glu–Met, γ-Glu–Leu, γ-Glu–Cys, and GSH were not transported by Seo1p. Investigating the specificity of plant γ-glutamyl dipeptide transporters is greatly needed for understanding the movement and function of these dipeptides in plants.

Additionally, more work is needed to determine if other non-proteinogenic dipeptides exist in plants. Plants contain hundreds of non-proteinogenic amino acids with known roles in growth and survival (Jander *et al.*, 2020), suggesting that they may also harbor many functional non-proteinogenic dipeptides. Studies in red algae have discovered citrullinylarginine that acts as a nitrogen store for winter months (Laycock *et al.*, 1981), but no further work has been done. Overall, non-proteinogenic dipeptides open up promising avenues for targeted stress resilience in plants by acting as microbial modulators, nitrogen stores, antioxidants, and potentially protein regulators.

## Cyclic dipeptides—to the rescue

Cyclic dipeptides, also known as DKPs, result from the intramolecular cyclization of linear dipeptides—a seemingly minor structural transformation that significantly alters their physicochemical and biological properties (Ortiz and Sansinenea, 2017; Bushman *et al.*, 2023; Jia *et al.*, 2023). These compact molecules exhibit high chemical and enzymatic stability, enhanced membrane permeability, and improved binding affinity for biological targets (Feni *et al.*, 2020), making them attractive scaffolds for drugs and agrochemicals. The biosynthesis of DKPs, however, is organism dependent and reveals intriguing gaps in our understanding, especially in plants. In bacteria, DKPs are produced enzymatically via well-characterized pathways involving cyclodipeptide synthetases (CDPSs), which utilize aminoacyl-tRNAs to construct cyclic scaffolds (Jacques *et al.*, 2015; Skinnider *et al.*, 2018), whereas fungi rely on non-ribosomal protein synthetases (NRPSs) (Gu *et al.*, 2013). In contrast, only a handful of DKPs were identified as endogenous metabolites produced by plants and animals, the notable example being cyclo(L-His–L-Pro) (Prasad *et al.*, 1977; Minen *et al.*, 2025). Plants lack the homologs of microbial CDPSs; hence, to produce cyclo(His–Pro) and possibly other yet to be identified DKPs, they are likely to exploit alternative routes. Plausible mechanisms include the spontaneous cyclization of dipeptides formed during protein turnover (Battersby *et al*., 1994) or the involvement of as yet uncharacterized plant enzymes that could utilize aminoacyl-tRNAs as substrates, similar to bacterial CDPSs (Fig. 1).

Despite uncertainties surrounding their biosynthesis, DKPs, mainly of microbial origin, have been identified as having physiological effects in plants, notably emulating endogenous hormone activity. The evidence that DKPs can mimic hormone activity is detailed in a recent study by Zhang *et al.* (2025). The study demonstrated that cyclo(L-Leu–L-Pro), produced by *Exiguobacterium* R2567 in the rice root microbiota, is transported to axillary buds where it binds to the strigolactone receptor OsD14. This binding event initiates strigolactone signaling, resulting in the degradation of OsD53 and the suppression of tiller outgrowth (see Box 1C). Similarly, a study by Yin *et al.* (2022) found that the application of 40 μM cyclo(L-Pro–L-Val) and cyclo(L-Pro–L-Tyr) enhances the interaction between the auxin receptor TIR1 and its co-repressors IAA7 and IAA17, facilitating their degradation and thereby releasing auxin response factors (ARFs). This molecular cascade leads to the transcriptional activation of auxin-responsive genes, including *LBD16/19* and *EXP4/14*, promoting the formation of lateral roots and root hairs via the downstream auxin-responsive gene *ARF7/19*. This mechanism supports earlier findings that ∼30 μM Pro-containing DKPs, such as cyclo(L-Pro–L-Tyr) and cyclo(L-Pro–L-Val), can mimic auxin activity to stimulate root branching and boost shoot biomass (Ortiz-Castro *et al.*, 2011; Gonzalez-Lopez *et al.*, 2021). Additionally, DKPs have been linked to the activation of TOR kinase signaling. Exogenous application of an ∼20 μM DKP mixture, comprising cyclo(L-Pro–L-Val), cyclo(L-Pro–L-Leu), and cyclo(L-Pro–L-Tyr) extracted from *Pseudomonas aeruginosa* PAO1 strain cultures in maize, resulted in increased phosphorylation of S6 kinase (S6K1), a canonical downstream effector of TOR (Corona-Sanchez *et al.*, 2019). DKP-treated seedlings exhibited significantly increased lateral and seminal root development and enhanced root surface area and volume, accompanied by dose-dependent phosphorylation of ZmS6K—indicative of TOR activation. In Arabidopsis, a similar effect was observed: an exogenous mixture of DKPs activated the TOR signaling pathway, leading to S6K1 phosphorylation, up-regulation of the mitotic marker gene *CYCB1*, and enhanced cell proliferation in root meristems—collectively promoting lateral root formation and biomass accumulation in a dose-dependent manner (Gonzalez-Lopez *et al.*, 2021). Thus, it could be accurate to say that DKPs are small molecules capable of mimicking the activity of endogenous hormones under specific conditions.

Besides growth regulation, DKPs can act as immune modulators capable of priming plant defenses against pathogens. For example, ∼10 µM cyclo(L-Gly–L-Pro), cyclo(L-Ala–L-Ile), cyclo(L-Ala–L-Leu), and cyclo(L-Leu–L-Pro) have been shown to activate induced systemic resistance (ISR) against *Pseudomonas syringae* in Arabidopsis without causing direct toxicity (Noh *et al*., 2017). These DKPs may function via jasmonic acid- (JA) and ethylene-dependent signaling, consistent with ISR-inducing plant growth-promoting rhizobacterial mechanisms. This assumption was later elucidated by Solis-Ortiz *et al*. (2022), who demonstrated that Arabidopsis seedlings previously exposed to ∼5 µM of the DPK isolate from the PAO1 culture are subsequently less affected by infection with pathogenic strains of *P. aeruginosa.* The DKP mix as well as the individual DKPs, cyclo(L-Pro–L-Leu), cyclo(L-Pro–L-Val), cyclo(L-Pro–L-Leu), and cyclo(L-Pro–L-Phe), induced expression of the *LOX2* gene, a well-established marker for JA activation (see Box 1D). Another relevant example is cyclo(L-Pro–L-Ser), which has been reported to provide antifungal protection in rice by disrupting the membrane integrity of *Fusarium* species (*F. verticillioides* and *F. fujikuroi*), having similar effectiveness to carbendazim, a commercial fungicide (Poonia *et al*., 2022). Together, these findings underscore the potential of DKPs as versatile agents in promoting plant health by modulating growth and immunity.

More than being immune modulators, DKPs were also shown to improve plant resilience to abiotic stressors. A recent study by Hung *et al*. (2024) reports the identification of cyclo(L-Ala–L-Gly) produced by the endophytic strain *Priestia megaterium* BP01R2, which was isolated from the Taiwanese salt marsh (*Bolboschoenus planiculmis*) plant. Cyclo(L-Ala–L-Gly) was among 18 mainly Pro-containing DKPs produced by BP01R2 in the presence of salt, and external supplementation of cyclo(L-Ala–L-Gly) was shown to alleviate the root growth penalty associated with salt stress in Arabidopsis (see Box 1E). However, the mode of action of cyclo(L-Ala–L-Gly) has yet to be established. Supporting the role of DPKs in plant resilience, Minen *et al.* (2025) demonstrated that cyclo(L-His–L-Pro) accumulation in Arabidopsis is stress and stage specific, with an *in planta* concentration of ∼1.5 µM in plants exposed to salt and ∼5 µM in older leaves (see Box 1F). In addition, cyclo(L-His–L-Pro) inhibited GAPC1, redirecting carbon from glycolysis to the PPP. As a result, cyclo(L-His–L-Pro) supplementation increased NADPH levels, enhancing the NADPH/NADP^+^ ratio. These findings are reminiscent of Tyr–Asp inhibition of GAPC1, discussed above, and raise the question of whether the mechanism of inhibition and downstream effects are the same. This can be addressed by elucidating the binding sites for each dipeptide and characterizing physiological changes in dipeptide-treated plants. Additionally, even if they exhibit the same function, it is important to know if there is an advantage of DKPs over linear dipeptides, such as the biological stability of the DKPs. By and large, DPKs represent attractive compounds for future studies using natural compounds to enhance stress resilience.

## Outlook

To date, dipeptides are a unique class of compounds that remain overlooked and underutilized, despite offering tremendous potential. Proteinogenic and non-proteinogenic dipeptides are present at basal levels in all organisms, typically reported in concentrations ranging from nanomolar to micromolar, as proteolysis, aging, and GSH cycling are regular processes, while cyclodipeptides accumulate only under specific growth and environmental conditions. However, they are all united by their marked increase in stress conditions, and recent work has identified specific dipeptides as promising compounds for improving plant health. However, many questions remain. These can be broadly categorized into (i) identification, (ii) function, and (iii) biogenesis. Starting with identification, it is important to determine the ‘who, where, and when’ of dipeptide interactions. We anticipate that the known dipeptides constitute just the tip of the iceberg present in nature and hidden in the dark matter of organismal metabolomes. The amino acid constituents, cyclization, and chemical modifications of the dipeptide scaffold contribute to the extensive structural diversity. Understanding how dipeptides accumulate in space and time is becoming increasingly accessible with the progress in metabolomics, especially their annotation through expanding compound libraries and online tools such as GNPS. Next, it is important to understand the ‘why’; that is, the function of dipeptides. As with all metabolites, a single dipeptide can have multiple functionalities, which can differ even between structurally similar dipeptides, such as retro dipeptides. Assigning a function is usually done by modulating compound levels by either direct supplementation or genetic engineering of metabolic pathways, followed by detailed phenotypic and molecular analysis. An online database of food-derived peptides, BIOPEP-UWM™, with *in silico*-predicted functions of dipeptides, serves as a framework for uncovering their biological roles *in vivo* (Minkiewicz *et al*., 2019). These methods, combined with techniques utilizing chromatography-MS such as PROMIS, MIDAS, and LiP-SMap (Liu and Gao, 2023), should be applied to identify and characterize dipeptide–protein interactions.

The ‘how’ concerns dipeptide biogenesis. As depicted in Fig. 1, there is a general understanding of dipeptide formation through protein degradation or enzymatic pathways. However, much is yet to be discovered. Do proteinogenic dipeptides have biosynthetic pathways? Are linear proteinogenic and non-proteinogenic dipeptides cyclized into cyclodipeptides in the plant? Can both linear and cyclic dipeptides be further chemically modified? As demonstrated in the previous sections, all dipeptides can accumulate in a specific manner, but what drives the specificity is unknown. Understanding the biosynthesis of dipeptides is crucial for bridging the gap between discovery and application for sustainable agriculture. Given the urgent need for environmentally friendly strategies to boost plant yields under increasingly harsh climates, we anticipate that the diverse plant health-promoting activities of dipeptides will make them excellent leads for next-generation agrochemicals. To summarize, we postulate that dipeptides constitute nature’s hidden gems.

## Data Availability

This is a review, no new data are being reported.
